# Healthy Eating Index-2015 and Predicted 10-Year Cardiovascular Disease Risk, as Well as Heart Age

**DOI:** 10.3389/fnut.2022.888966

**Published:** 2022-07-12

**Authors:** Yinyin Zhang, Cong Lu, Xinyu Li, Yameng Fan, Jiaqiao Li, Yamei Liu, Yan Yu, Long Zhou

**Affiliations:** ^1^School of Public Health, Xi’an Jiaotong University, Xi’an, China; ^2^Department of Cardiology, Sichuan Provincial People’s Hospital, University of Electronic Science and Technology of China, Chengdu, China

**Keywords:** HEI-2015, 10-year CVD risk, heart age, cross-sectional study, NHANES

## Abstract

**Background and Aims:**

Dietary factor plays an important role in the prevention of cardiovascular disease (CVD). The healthy eating index-2015 (HEI-2015), an indicator of the overall dietary quality, has been introduced to reflect adherence to the 2015–2020 Dietary Guidelines for Americans (DGA). This study aims to explore the associations of the HEI-2015 with predicted 10-year CVD risk and heart age among United States adults aged 30–74 years old using data from the National Health and Nutrition Examination Survey (NHANES) 2011–2014.

**Methods and Results:**

We conducted a cross-sectional analysis among 6,614 participants aged 30–74 years old. The HEI-2015 scores were calculated from 2-days 24-h dietary recall interviews. The 10-year CVD risk and heart age were derived from the sex-specific Framingham general cardiovascular disease risk score. We defined high cardiovascular disease risk as a predicted 10-year cardiovascular disease risk of > 20%. Multiple linear regression and binary logistic regression models were used to investigate the associations of the HEI-2015 with predicted 10-year CVD risk and heart age. Compared with participants in the lowest HEI-2015 quartile, those in the highest quartile had lower predicted 10-year CVD risk (β = −2.37, 95% CI: −3.09 to –1.65, *P* < 0.0001), lower heart age (β = −2.63, 95% CI: −3.29 to –1.96, *P* < 0.0001) and lower odds for high risk of CVD (OR = 0.62, 95% CI: 0.49 to 0.80, *P*-trend < 0.0001) after adjusting for multiple covariates.

**Conclusion:**

Higher adherence to the 2015–2020 Dietary Guidelines for Americans is associated with lower predicted 10-year cardiovascular disease risk and lower heart age among United States adults.

## Introduction

Cardiovascular disease (CVD) remains the leading cause of death in the United States in 2018, with coronary heart disease (CHD) leading the list (42.1%), followed by stroke (17%), hypertension (11%), heart failure (9.6%), and arterial disease (2.9%) (1). Age, sex, high blood pressure, smoking, high cholesterol, and diabetes are recognized as risk factors for CVD (1–4). To help with the primary prevention of persons at high CVD risk, D’Agostino et al. developed a model based on the Framingham Heart Study to assess a person’s absolute risk of developing CVD in the next 10 years. This general CVD risk prediction model was well-discriminative and calibrated, with a C statistic ranging from 0.76 to 0.79. The C statistic is similar to the area under the receiver operating characteristic curve (AUC) and it is usually used to reflect the predictive value for a prediction model (5). The predicted 10-year CVD risk score is useful for clinicians to provide treatment recommendations to patients with CVD and for patients to conduct self-cardiac assessments based on this model (6). As a new concept derived from the Framingham risk score, heart age was defined as the chronological age of a person with the same CVD risk score but other risk factors at the normal level (5). Compared with 10-year CVD risk, heart age is more likely to prompt and motivate people to understand CVD risk factors and improve life behaviors to arouse emotional resonance (7, 8).

According to the global burden of disease study, the health care cost of CVD continues to rise in recent years (9, 10). The direct cost of CVD in the United States was estimated to be as high as $216 billion in 2016–2017, while cancer costs only $105.6 billion (11). Improving unhealthy dietary behaviors provides crucial new insights into reducing the risk of CVD and alleviating the healthcare burden caused by CVD (12, 13). The Healthy Eating Index (HEI) is updated every 5 years as a comprehensive measure of diet quality. HEI-2015, the latest version of the HEI, was developed to evaluate adherence to the 2015–2020 Dietary Guidelines for Americans (DGA) (14). To date, few studies have investigated the association between the HEI-2015 score and CVD risk in the general population (15–17). In this study, we provide the most recent estimates of the associations between HEI-2015 and predicted 10-year CVD risk, as well as heart age, in a representative United States population aged 30–74 years old, based on combined data from the 2011–2012 and 2013–2014 National Health and Nutrition Examination Surveys (NHANES).

## Materials and Methods

### Study Population

**FIGURE 1:**
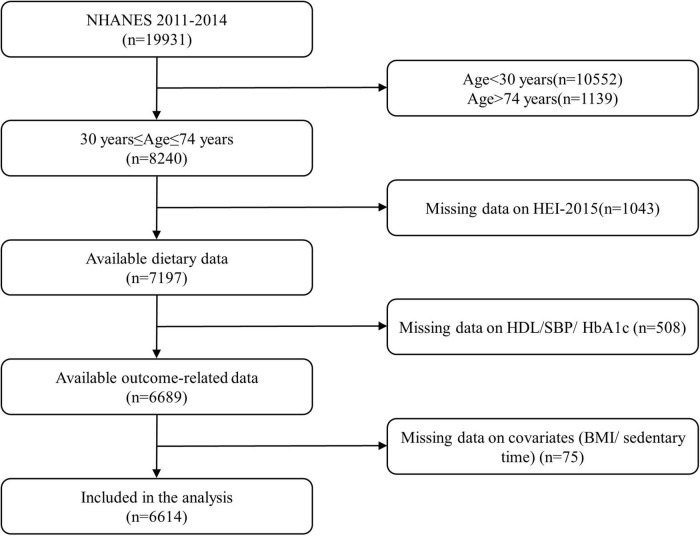
Flow diagram of the study participant selection. NHANES, National Health and Nutrition Examination Survey; HEI-2015, Healthy Eating Index-2015; HDL, high-density lipoprotein; SBP, systolic blood pressure; HbA1c, glycated hemoglobin A1c; BMI, body mass index.

National Health and Nutrition Examination Surveys is a nationally representative cross-sectional survey conducted by the National Center for Health Statistics (NCHS) of the Centers for Disease Control and Prevention (CDC) (18). The Framingham general CVD risk score was recommended for use in the 30–74 age range (5). Of the participants who completed the interview (19,931 in the NHANES 2011–2014), we included 8,240 participants aged 30–74 in this study. We further excluded participants with missing HEI-2015 data (*n* = 1,043), missing data for variables for the construction of 10-year CVD risk score [high-density lipoprotein cholesterol (HDL-C), systolic blood pressure (SBP), and glycated hemoglobin A1c (HbA1c)] (*n* = 508), and missing data on covariate (*n* = 75). A total of 6,614 participants were included in the final analysis. A flow chart of the sample selection is shown in Figure 1.

### Healthy Eating Index-2015 Assessment

The dietary interview component of the National Health and Nutrition Examination Survey was collected for What We Eat in America (WWEIA). WWEIA is conducted as a partnership between the United States Department of Agriculture (USDA) and the United States Department of Health and Human Services (DHHS). DHHS is responsible for the survey sample design and data collection, while USDA is responsible for the dietary data collection methodology, maintenance of the databases used to code and process the data, and data review and processing. The USDA Automated Multiple-Pass Method (AMPM) is used for collecting 24-h dietary recalls in WWEIA (19). The first day was conducted face-to-face in the dietary interview room of the Mobile Examination Center (MEC) and the second day was collected *via* telephone 3–10 days later. The food group of the diet calculation was determined by the USDA Food Patterns Equivalence Database, and energy or nutrient content was determined by the USDA Food and Nutrient Database for Dietary Studies.

The calculation of the HEI-2015 score was not based on the absolute amount of ingredients but was based on the energy density per 1,000 kcal (except fatty acids). Fatty acids were scored as unsaturated fatty acids divided by saturated fatty acids. The index with a total score ranging from 0 to 100 consists of 13 components: total fruits, whole fruits, total vegetables, greens and beans, whole grains, dairy, total protein foods, seafood and plant proteins, fatty acids (nine recommended components to include in a healthy diet) and refined grains, sodium, added sugars, and saturated fats (four components that should be consumed sparingly). Each component is scored separately and added together to obtain the HEI-2015 total score. Adequacy components intake is proportional to score, while the moderation components are the opposite.

### Estimation of the 10-Year Cardiovascular Disease Risk and Heart Age

The endpoint used in the Framingham Heart Study for predicting CVD risk was a composite outcome including CHD (coronary death, myocardial infarction, coronary insufficiency, and angina), cerebrovascular events (including ischemic stroke, hemorrhagic stroke, and transient ischemic attack), peripheral artery disease (intermittent claudication), and heart failure (20). The sex-specific Framingham general CVD risk score is based on age, sex, total cholesterol (TC), HDL-C, SBP, hypertension treatment or not, diabetes mellitus, and smoking status (5). Regarding the CVD risk, the population was further stratified into two risk categories: low (predicted 10-year CVD risk score of ≤ 20%) and high (predicted 10-year CVD risk score of > 20%) (5). Meanwhile, heart age, defined as the age of a person with the same predicted risk of CVD but with all other risk factors at the normal level, was also estimated using the sex-specific-based Framingham general CVD risk score, and the risk factors used in the calculation were fully consistent with the predicted 10-year CVD risk. Briefly, we first calculated the CVD points for men and women based on the CVD risk factors mentioned above. Then, the predicted heart age was determined by converting the CVD points according to the “heart age” sheet published previously (5).

### Definition of Covariates

Sociodemographic, lifestyle factors, and physical examination data were obtained through household questionnaires by bilingual trained study staff. The self-reported demographic covariates included sex (male or female), educational level (<high school, high school, or > high school), ethnicity (Hispanic, non-Hispanic White, non-Hispanic Black, or others), and family economic situation (income-to-poverty ratio ≤ 1.30, 1.31–1.85, and > 1.85). The family monthly poverty level index (income-to-poverty ratio) represents household income as a ratio of total family income to the poverty level defined by the Department of Health and Human Services (DHHS).

As for lifestyle factors, smokers were defined as those who had smoked at least 100 cigarettes in life. Drinkers were defined as participants who consumed alcohol 12 or more times in any given year. Sedentary time is an indicator of sedentary behavior refers to the amount of time spent sitting during the day other than sleep.

All anthropometric data were measured by highly trained medical personnel to minimize errors in the measurement process. SBP and diastolic blood pressure were the averages of three consecutive readings of measurements in a sitting position after a 5-min quiet rest. A fourth blood pressure measurement was taken if one reading was incomplete. Standing height was measured by a stadiometer and body weight was measured by a digital weight scale requiring participants to wear a standard examination gown and no jewelry. Body mass index (BMI) (kg/m^2^) was defined as weight (kg) divided by height squares (m^2^).

Diabetes was diagnosed as HbA1c ≥ 6.5% or currently using insulin or diabetes medications. Tosoh Automated Glycohemoglobin Analyzer HLC-723G8 was used to determine the content of % HbA1c in whole blood (21). The analysis of serum HDL-C and serum TC was carried out by Roche/Hitachi Modular P Chemistry Analyzer, for measurement of HDL-C by a magnesium/dextran sulfate method and serum TC by a completely enzymatic method (22, 23).

### Statistical Analysis

Considering the complex sampling design and multi-year data of NHANES 2011–2014, the sample weights for both cycles were used for statistical analysis. The results are presented as weighted mean ± SE for continuous variables and weighted percentages for categorical variables. To compare characteristics by HEI-2015 quartile groups, we conducted weighted one-way analyses of variance for continuous variables and weighted chi-square tests for categorical variables. The associations of HEI-2015 with 10-year CVD risk and heart age were estimated using multivariable linear regression models according to quartiles of the HEI-2015, in which the lowest quartile was used as the reference category. The binary logistic regression models were also used to determine the odds ratio (OR) and 95% confidence interval (CI) for the high predicted 10-year risk of CVD, with adjusting for potential confounders. We also used the HEI-2015 score as a continuous variable in the analysis. Model 1 adjusted for age and sex. Model 2 additionally adjusted for ethnicity, drinking status, education level, family monthly poverty level, and sedentary time. In Model 3, we adjusted for all the covariates in Model 2 plus BMI as a continuous variable. To test the overall trend, we used the median of HEI-2015 in each quartile as a continuous variable in the regression model. In addition, the interactions between HEI-2015 and various covariates have also been tested, and stratified analyses were performed. Statistical significance for all analyses was two-tailed *P* < 0.05. All analyses were performed using SAS version 9.4 (SAS Institute, Cary, NC, United States).

## Results

### Characteristics of Study Participants

We identified 6,614 participants with sufficient information to predict the 10-year CVD risk (3,211 men and 3,403 women). The general characteristics of the participants are shown in Table 1. Participants with higher HEI-2015 scores were more likely to be older, female, more likely to have higher educational level and family monthly poverty level index, and less likely to be a drinker. In contrast, weighted mean BMI was higher among participants who had a lower HEI-2015 score. No statistically significant differences were found between groups for the variable of sedentary time.

**TABLE 1:** Characteristics of study participants according to HEI-2015 quartile (weighted analysis).^1^

Characteristic	Quartile of HEI-2015				*P*-value^2^
	Q1 (19.0–44.8)	Q2 (44.8–54.4)	Q3 (54.4–64.4)	Q4 (64.4–95.8)	
*N*	1,653	1,654	1,654	1,653	
Age (years)	47.5 ± 0.3	50.0 ± 0.3	50.5 ± 0.3	52.9 ± 0.3	<0.0001
**Sex, n (%)**					<0.0001
Male	889 (54.4)	840 (52.7)	789 (47.3)	693 (40.5)	
Female	764 (45.6)	814 (47.3)	865 (52.7)	960 (59.5)	
**Ethnic, n (%)**					<0.0001
Hispanic	306 (12.5)	367 (14.0)	413 (14.8)	382 (12.5)	
Non-Hispanic white	778 (69.7)	665 (68.4)	628 (67.9)	605 (70.0)	
Non-Hispanic black	431 (12.8)	415 (11.5)	376 (10.4)	295 (7.5)	
Other races	138 (5.1)	207 (6.2)	237 (6.9)	371 (9.9)	
**Education, n (%)**					<0.0001
<High school	434 (20.2)	371 (15.2)	361 (14.9)	253 (8.9)	
High school	428 (25.8)	415 (24.7)	315 (16.3)	262 (14.0)	
>High school	791 (54.0)	868 (60.1)	978 (68.8)	1,138 (77.1)	
**Family monthly poverty level category, n (%)**					<0.0001
≤1.30	720 (31.9)	580 (24.9)	510 (20.1)	371 (14.5)	
1.31–1.85	274 (14.5)	253 (11.9)	285 (14.1)	252 (10.6)	
>1.85	659 (53.6)	821 (63.2)	859 (65.8)	1030 (74.9)	
**Drinker, n (%)**					<0.0001
Yes	1,214 (77.7)	1,172 (77.4)	1,148 (77.1)	1,103 (75.2)	
No	439 (22.3)	482 (22.6)	506 (22.9)	550 (24.9)	
BMI (kg/m^2^)	30.4 ± 0.2	30.4 ± 0.2	29.3 ± 0.2	27.9 ± 0.1	<0.0001
Sedentary time (h/day)	6.6 ± 0.1	6.7 ± 0.1	6.8 ± 0.1	6.8 ± 0.1	0.3891

*^1^Values are presented as weighted mean ± SE or number (weighted %); HEI-2015, healthy eating index-2015; BMI, body mass index; Q, quartile; Q1 refer to the unhealthiest diet quality; Q4 refer to the healthiest diet quality.*

*^2^Weighting factors were used in calculating P-values to account for the complex survey design of NHANES. One-way analyses of variance were used for continuous variables and the chi-square test was used for categorical variables.*

### The Association of the HEI-2015 Score With Predicted 10-Year Cardiovascular Disease Risk and Heart Age

Results of multivariable linear regression analyses for the association of HEI-2015 score with 10-year CVD risk and heart age are shown in Table 2. There were significant negative associations of HEI-2015 with 10-year CVD risk and heart age after adjusting for age, sex, ethnicity, drinker, education level, family monthly poverty level, sedentary time, and BMI. The corresponding regression coefficients of the highest quartile group of the HEI-2015 score were −2.05 (95% CI: −2.76 to –1.33, *P* < 0.0001) for 10-year CVD risk and −2.19 (95% CI: −2.85 to –1.53, *P* < 0.0001) for heart age, with the lowest quartile group as the reference. In addition, linear trends were also observed in the associations of HEI-2015 with 10-year CVD risk (*P*-trend<0.05) and heart age (*P*-trend<0.05).

**TABLE 2:** Regression coefficients and 95% confidence intervals of HEI-2015 for 10-year CVD risk and heart age.^1^

HEI-2015	Predicted 10-year CVD risk			Heart age		
	Unstandardized coefficients B (95%CI)	*P*-value	*P* trend^5^	Unstandardized coefficients B (95%CI)	*P*-value	*P* trend
**Model1^2^**						
HEI-2015^6^	−0.09 (−0.10, −0.07)	<0.0001		−0.10 (−0.11, −0.08)	<0.0001	
Q1	0 (reference)	–	<0.0001	0 (reference)	–	<0.0001
Q2	−0.44 (−1.14, 0.25)	0.2110		−0.85 (−1.49, −0.21)	0.0096	
Q3	−1.46 (−2.15, −0.76)	<0.0001		−1.76 (−2.41, −1.12)	<0.0001	
Q4	−3.13 (−3.83, −2.43)	<0.0001		−3.48 (−4.14, −2.83)	<0.0001	
**Model2^3^**						
HEI-2015	−0.07 (−0.08, −0.05)	<0.0001		−0.07 (−0.09, −0.06)	<0.0001	
Q1	0 (reference)	–	<0.0001	0 (reference)	–	<0.0001
Q2	−0.18 (−0.87, 0.51)	0.6160		−0.57 (−1.21, 0.07)	0.0791	
Q3	−1.08 (−1.78, −0.38)	0.0023		−1.31 (−1.95, −0.66)	<0.0001	
Q4	−2.37 (−3.09, −1.65)	<0.0001		−2.63 (−3.29, −1.96)	<0.0001	
**Model3^4^**						
HEI-2015	−0.06 (−0.07, −0.04)	<0.0001		−0.06 (−0.08, −0.04)	<0.0001	
Q1	0 (reference)	–	<0.0001	0 (reference)	–	<0.0001
Q2	−0.15 (−0.83, 0.53)	0.6664		−0.54 (−1.17, 0.09)	0.0934	
Q3	−0.92 (−1.61, −0.23)	0.0093		−1.09 (−1.72, −0.45)	0.0008	
Q4	−2.05 (−2.76, −1.33)	<0.0001		−2.19 (−2.85, −1.53)	<0.0001	

*^1^HEI-2015, healthy eating index-2015; Q, quartile; Q1 refers to the unhealthiest diet quality; Q4 refers to the healthiest diet quality; B, unstandardized regression coefficient; CI, confidence interval. Unstandardized coefficients B and 95% CI from Multivariable linear regression models with adjustment as follows:*

*^2^Model 1 adjusted for age, and sex.*

*^3^Model 2 adjusted for variables in model1 + ethnicity, drinker, education level, family monthly poverty level, and sedentary time.*

*^4^Model 3 adjusted for variables in model 2 + BMI.*

*^5^P trend, Test for trend was a sequential test of the quartiles of dietary quality scores.*

*^6^HEI-2015 as a continuous variable.*

### The Association of HEI-2015 With High 10-Year Cardiovascular Disease Risk Among United States Adults Aged 30–74 Years Old

**FIGURE 2:**
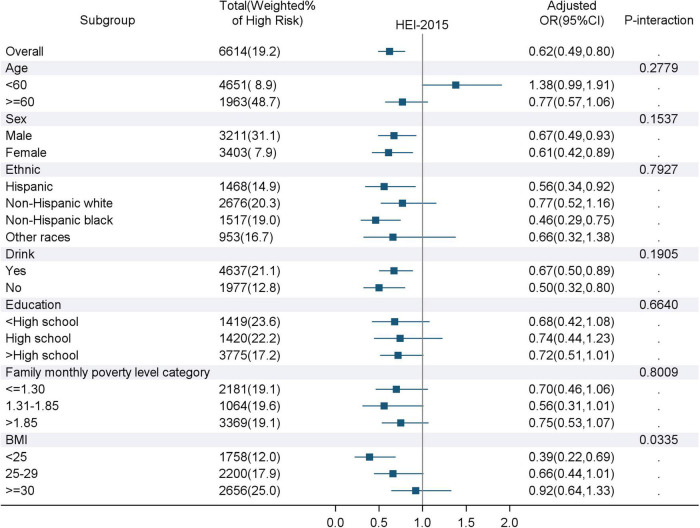
Associations of the highest quartile of HEI-2015 score with high predicted 10-year CVD risk for subgroups. HEI-2015, Healthy Eating Index-2015; BMI, Body Mass Index; OR, odds ratio; CI, confidence interval. The variables adjusted for in the subgroup analysis were consistent with Model 3 in Table 3, except for the subgroup variables that were not included in the model. P-interaction refers to the interaction analysis between HEI-2015 and various covariates.

The ORs and 95% CIs for high 10-year CVD risk (predicted 10-year risk > 20%) are presented in Table 3. In the age and sex-adjusted model (model 1), participants who had the highest quartile of HEI-2015 score had lower odds of high 10-year CVD risk compared to those who had the lowest quartile of HEI-2015 score (OR: 0.51; 95%CI: 0.40–0.64; *P*-trend<0.0001). The significant association remained when further it is adjusted for ethnicity, drinking status, education level, family monthly poverty level, sedentary time, and BMI, and the corresponding OR (95% CI) was 0.62 (0.49–0.80). In addition, the interactions between other covariates and HEI-2015 scores, except for BMI (P for interaction = 0.0335), were not significant (Figure 2). The variables adjusted for subgroup analysis in Figure 2 were consistent with Model 3 in Table 3, except for the subgroup variables that were not included in the model. A subgroup analysis according to normal or wasting (BMI < 25 kg/m^2^), overweight (25 ≤ BMI < 30 kg/m^2^), and obese (BMI ≥ 30 kg/m^2^) showed that participants with normal BMI and the highest HEI-2015 score had 61% lower odds of high 10-year CVD risk (OR = 0.39, 95% CI: 0.22–0.69) compared with those with the lowest HEI-2015 score.

**TABLE 3:** Odds ratios (OR) and 95% confidence intervals (CI) of HEI-2015 for high predicted 10-year CVD risk.^1^

	HEI-2015					*P* trend^5^
	Continuous^6^	Q1	Q2	Q3	Q4	
Total	6,614	1,653	1,654	1,654	1,653	
No of participants at high risk (>20%)	1,474	362	412	356	344	
Model 1^2^	0.98 (0.97, 0.99)	1 (reference)	1.08 (0.86, 1.36)	0.69 (0.54, 0.86)	0.51 (0.40, 0.64)	<0.0001
Model 2^3^	0.984 (0.98, 0.99)	1 (reference)	1.14 (0.91, 1.44)	0.73 (0.57, 0.92)	0.59 (0.47, 0.76)	<0.0001
Model 3^4^	0.986 (0.98, 0.99)	1 (reference)	1.13 (0.89, 1.42)	0.75 (0.59, 0.95)	0.62 (0.49, 0.80)	<0.0001

*^1^HEI-2015, healthy eating index-2015; Q, quartile; Q1 refer to the unhealthiest diet quality; Q4 refer to the healthiest diet quality. OR and 95% CI from a binary logistic regression model with adjustment as follows:*

*^2^Model 1 adjusted for age, and sex.*

*^3^Model 2 adjusted for variables in model 1 + ethnicity, drinker, education level, family monthly poverty level, and sedentary time.*

*^4^Model 3 adjusted for variables in model 2 + BMI.*

*^5^P trend, Test for trend was a sequential test of the quartiles of dietary quality scores.*

*^6^ HEI-2015 as a continuous variable.*

## Discussion

In this cross-sectional study, we found that a higher HEI-2015 score was associated with a significantly lower predicted 10-year CVD risk and lower heart age in United States general population aged 30–74 years old.

Numerous studies have shown that some foods and dietary ingredients were associated with the risk of CVD (24–27), and those studies usually draw inconsistent conclusions. For example, several studies found that saturated fatty acid (SFA) intake was positively associated with cardiovascular events (28–30), but a recent review reported that there is a lack of association between SFA consumption and non-communicable diseases (31). Although the research on the effect of a single food or nutrient on CVD risk has extensive and profound significance, people’s daily diet is a mixture of multiple foods and nutrients. In recent years, the impact of complete dietary patterns on CVD has aroused widespread interest, such as the Mediterranean diet (MedDiet), the Dietary Approach to Stop Hypertension (DASH) diet, the vegetarian dietary pattern, etc. The Mediterranean diet, which is rich in whole grains, vegetables, fruits, and olive oil (replace SFA with USFA), etc., has strong relevance for reducing the incidence and mortality of CVD and preventing the occurrence of various chronic diseases (32–34). The DASH diet is generally used to reduce high blood pressure, one of the risk factors for CVD, and, thus, it also makes a significant contribution to the prevention of CVD (34–36). A systematic review and meta-analysis of the DASH diet have shown that there was a significant inverse linear relationship between DASH diet consumption and CVD risk, and the changes in blood pressure and cholesterol concentration caused by the DASH diet would reduce the predicted 10-year CVD risk by approximately 13% (37, 38). Some previous studies have examined the relationship between dietary patterns based on dietary guidelines and CVD, but most of them have focused on revealing the occurrence of specific cardiovascular outcome events (such as CHD, stroke) and CVD mortality (15–17). A recent analysis of the relationship between HEI-2015 and CVD based on the Nurses’ Health Study (NHS), NHS II, and Health Professionals Follow-up Study (HPFS) has shown that higher diet scores were significantly associated with a lower risk of cardiovascular events (15), which is similar to our findings. Our study population was more comprehensive and representative of the United States population of all races and genders than the study population, which included only female nurses and male health professionals. Our study of the HEI-2015 in the NHANES 2011–2014 is consistent with previous findings in the Multiethnic Cohort, linking HEI-2015 with reduced CVD risk (39). HEI-2015 has also demonstrated the predictive criterion validity for all-cause, cancer, and CVD mortality in the United States Participants, with the highest diet quality had a 13% to 21% decreased risk of CVD mortality compared with the lowest diet quality (40).

Apart from the aforementioned studies on CVD, only two studies focused on the relationship between dietary guideline-based dietary indices and predicted 10-year CVD risk and showed mixed results. A randomized study of Iranian male military personnel found no significant relationship between 10-year CVD risk and the Healthy Diet Indicator (HDI)-2020 (41). HDI-2020, based on the Global Dietary Recommendations (GDR), is the latest version of the WHO publication for the prevention of chronic diseases, and its components are calculated as a percentage of energy (42). Results from the randomized sample study of Iranian employees also showed no significant association between the Alternative Healthy Eating Index (AHEI) and predicted 10-year CVD risk (43). The AHEI is also designed to reduce food and nutrient intake associated with chronic disease risk (44). Different from the above two studies, our study used HEI-2015, an index reflecting the dietary guidelines for United States residents, in a nationally representative United States population. We found that there was a significant inverse association between HEI-2015 and the predicted 10-year CVD risk (OR: 0.62; 95%CI: 0.49–0.80; *P*-trend<0.0001). Furthermore, similar results were also found when we replaced predicted 10-year CVD risk with heart age as the outcome in the analysis. Compared with 10-year CVD risk, the psychological impact of the concept of heart age on the general population will be more intuitive.

There are several strengths in our study. First, we used the latest version of HEI to explore the relationship between the overall diet quality and the predicted 10-year CVD risk. HEI-2015, a comprehensive dietary index, can better reflect Americans’ compliance with the 2015–2020 DGA. Second, NHANES provides a large and nationally representative sample of United States adults. However, our study has several limitations. First, two self-reported 24-h dietary interviews recall data maybe not be a good indicator to reflect the long-term diet consumption. Second, dietary data were subject to recall bias as participants self-reported their diet through 2 days of 24-h dietary recall interviews. Third, cross-sectional studies cannot accurately reflect the causal relationship between the two variables. Fourth, although we adjusted for many potential confounders, we could not rule out the effect of confounders not measured in this study.

## Conclusion

In conclusion, we found that HEI-2015 total scores following the 2015–2020 Dietary Guidelines for Americans were negatively associated with predicted 10-year CVD risk and heart age. Our study adds to the evidence of an association between the HEI-2015 total score and the risk of CVD and demonstrates the importance of following dietary guidelines for the prevention of CVD in the general United States population.

## Data Availability Statement

Publicly available datasets were analyzed in this study. This data can be found here: National Center for Health Statistics (NHANES): https://wwwn.cdc.gov/nchs/nhanes/Default.aspx.

## Author Contributions

YY, LZ, YZ, and CL contributed to the study design. LZ, YZ, and CL performed the data cleaning and analysis. YZ drafted the manuscript. LZ, XL, and YF critically revised and edited the manuscript for important intellectual content. All authors approved the final manuscript to be published.

## Conflict of Interest

The authors declare that the research was conducted in the absence of any commercial or financial relationships that could be construed as a potential conflict of interest. The reviewer XW declared a past collaboration with one of the authors LZ to the handling editor.

## Publisher’s Note

All claims expressed in this article are solely those of the authors and do not necessarily represent those of their affiliated organizations, or those of the publisher, the editors and the reviewers. Any product that may be evaluated in this article, or claim that may be made by its manufacturer, is not guaranteed or endorsed by the publisher.

## Abbreviations

CVD: cardiovascular disease
HEI: healthy eating index
DGA: Dietary Guidelines for Americans
NHANES: National Health and Nutrition Examination Survey
CHD: coronary heart disease
AUC: Area Under Curve
US: United States
NCHS: National Center for Health Statistics
CDC: Centers for Disease Control and Prevention
HDL-C: high-density lipoprotein cholesterol
SBP: systolic blood pressure
HbA1c: glycated hemoglobin A1c
WWEIA: What We Eat in America
USDA: United States Department of Agriculture
DHHS: United States Department of Health and Human Services
AMPM: Automated Multiple-Pass Method
MEC: Mobile Examination Center
TC: total cholesterol
HHS: Department of Health and Human Services
BMI: body mass index
SE: standard error
OR: odds ratio
CI: confidence interval
SFA: saturated fatty acid
MedDiet: Mediterranean diet
DASH: Dietary Approach to Stop Hypertension
USFA: unsaturated fatty acid
NHS: Nurses’ Health Study
HPFS: Health Professionals Follow-up Study
MEC: Multiethnic Cohort
HDI: Healthy Diet Indicator
GDR: Global Dietary Recommendations
AHEI: Alternative Healthy Eating Index
WHO: World Health Organization.

## References

[1] Virani SS Alonso A Aparicio HJ Benjamin EJ Bittencourt MS Callaway CW Heart disease and stroke statistics-2021 update: a report from the American heart association. *Circulation.* (2021) 143:e254–743. 10.1161/CIR.0000000000000950 33501848 PMC13036842

[2] Wilson PW D’Agostino RB Levy D Belanger AM Silbershatz H Kannel WB. Prediction of coronary heart disease using risk factor categories. *Circulation.* (1998) 97:1837–47. 10.1161/01.cir.97.18.1837 9603539

[3] Anderson KM Odell PM Wilson PWF Kannel WB. Cardiovascular-disease risk profiles. *Am Heart J.* (1991) 121:293–8. 10.1016/0002-8703(91)90861-B 1985385

[4] Grundy SM Balady GJ Criqui MH Fletcher G Greenland P Hiratzka LF Primary prevention of coronary heart disease: guidance from Framingham: a statement for healthcare professionals from the AHA task force on risk reduction. American heart association. *Circulation.* (1998) 97:1876–87. 10.1161/01.cir.97.18.1876 9603549

[5] D’Agostino RB Sr Vasan RS Pencina MJ Wolf PA Cobain M Massaro JM General cardiovascular risk profile for use in primary care: the Framingham heart study. *Circulation.* (2008) 117:743–53. 10.1161/CIRCULATIONAHA.107.699579 18212285

[6] Lonnberg L Ekblom-Bak E Damberg M. Reduced 10-year risk of developing cardiovascular disease after participating in a lifestyle programme in primary care. *Ups J Med Sci.* (2020) 125:250–6. 10.1080/03009734.2020.1726533 32077778 PMC7720946

[7] Soureti A Hurling R Murray P van Mechelen W Cobain M. Evaluation of a cardiovascular disease risk assessment tool for the promotion of healthier lifestyles. *Eur J Cardiovasc Prev Rehabil.* (2010) 17:519–23. 10.1097/HJR.0b013e328337ccd3 20195154

[8] Lopez-Gonzalez AA Aguilo A Frontera M Bennasar-Veny M Campos I Vicente-Herrero T Effectiveness of the heart age tool for improving modifiable cardiovascular risk factors in a Southern European population: a randomized trial. *Eur J Prev Cardiol.* (2015) 22:389–96. 10.1177/2047487313518479 24491403

[9] Abbafati C Abbas KM Abbasi M Abbasifard M Abbasi-Kangevari M Abbastabar H Global burden of 369 diseases and injuries in 204 countries and territories, 1990-2019: a systematic analysis for the global burden of disease study 2019. *Lancet.* (2020) 396:1204–22. 10.1016/S0140-6736(20)30925-9 33069326 PMC7567026

[10] Roth GA Mensah GA Johnson CO Addolorato G Ammirati E Baddour LM Global burden of cardiovascular diseases and risk factors, 1990-2019: update from the GBD 2019 study. *J Am Coll Cardiol.* (2020) 76:2982–3021. 10.1016/j.jacc.2020.11.010 33309175 PMC7755038

[11] Agency for Healthcare Research and Quality. Medical Expenditure Panel Survey (MEPS): Household Component Summary Tables: Medical Conditions, 2016 and Later. (2021). Available online at: https://catalog-prod-datagov.app.cloud.gov/dataset/medical-expenditure-panel-survey-household-component (accessed October 15, 2021).

[12] Mozaffarian D. Dietary and policy priorities for cardiovascular disease, diabetes, and obesity: a comprehensive review. *Circulation.* (2016) 133:187–225. 10.1161/CIRCULATIONAHA.115.018585 26746178 PMC4814348

[13] Yu E Malik VS Hu FB. Cardiovascular disease prevention by diet modification: JACC health promotion series. *J Am Coll Cardiol.* (2018) 72:914–26. 10.1016/j.jacc.2018.02.085 30115231 PMC6100800

[14] Krebs-Smith SM Pannucci TE Subar AF Kirkpatrick SI Lerman JL Tooze JA Update of the healthy eating index: HEI-2015. *J Acad Nutr Diet.* (2018) 118:1591–602. 10.1016/j.jand.2018.05.021 30146071 PMC6719291

[15] Shan Z Li Y Baden MY Bhupathiraju SN Wang DD Sun Q Association between healthy eating patterns and risk of cardiovascular disease. *JAMA Intern Med.* (2020) 180:1090–100. 10.1001/jamainternmed.2020.2176 32539102 PMC7296454

[16] Hu EA Steffen LM Coresh J Appel LJ Rebholz CM. Adherence to the healthy eating index-2015 and other dietary patterns may reduce risk of cardiovascular disease, cardiovascular mortality, and all-cause mortality. *J Nutr.* (2020) 150:312–21. 10.1093/jn/nxz218 31529069 PMC7373820

[17] Xu Z Steffen LM Selvin E Rebholz CM. Diet quality, change in diet quality and risk of incident CVD and diabetes. *Public Health Nutr.* (2020) 23:329–38. 10.1017/S136898001900212X 31511110 PMC6992481

[18] Centers for Disease Control and Prevention, National Center for Health Statistics. *National Health and Nutrition Examination Survey. About NHANES.* (2021). Available online at: https://www.cdc.gov/nchs/nhanes/about_nhanes.htm (accessed December 2, 2021).

[19] Moshfegh AJ Rhodes DG Baer DJ Murayi T Clemens JC Rumpler WV The US department of agriculture automated multiple-pass method reduces bias in the collection of energy intakes. *Am J Clin Nutr.* (2008) 88:324–32. 10.1093/ajcn/88.2.324 18689367

[20] Eaker ED Garrison RJ Castelli WP. Risk-factors for coronary heart-disease among women – 30 years of follow-up from the Framingham heart-study. *Circulation.* (1985) 71:A414.

[21] Centers for Disease Control and Prevention, National Center for Health Statistics. *National Health and Nutrition Examination Survey. Measurement Method of Nhanes Hemoglobin A1c(Glycohemoglobin).* (2021). Available online at: https://wwwn.cdc.gov/nchs/data/nhanes/2011-2012/labmethods/ghb_met_g_tosoh_g8.pdf (accessed December 2, 2021).

[22] Centers for Disease Control and Prevention, National Center for Health Statistics. *National Health and Nutrition Examination Survey. Measurement Method of Nhanes HDL-Cholesterol.* (2021). Available online at: https://wwwn.cdc.gov/nchs/data/nhanes/2011-2012/labmethods/hdl_g_met_hdl.pdf (accessed December 10, 2021).

[23] Centers for Disease Control and Prevention, National Center for Health Statistics. *National Health and Nutrition Examination Survey. Measurement Method of Nhanes Serum Total Cholesterol.* (2021). Available online at: https://wwwn.cdc.gov/nchs/data/nhanes/2011-2012/labmethods/tchol_g_met.pdf (accessed December 10, 2021).

[24] Wang DD Hu FB. Dietary fat and risk of cardiovascular disease: recent controversies and advances. *Annu Rev Nutr.* (2017) 37:423–46. 10.1146/annurev-nutr-071816-064614 28645222

[25] Astrup A Magkos F Bier DM Brenna JT de Oliveira Otto MC Hill JO Saturated fats and health: a reassessment and proposal for food-based recommendations: JACC state-of-the-art review. *J Am Coll Cardiol.* (2020) 76:844–57. 10.1016/j.jacc.2020.05.077 32562735

[26] Mente A O’Donnell M Rangarajan S Dagenais G Lear S McQueen M Associations of urinary sodium excretion with cardiovascular events in individuals with and without hypertension: a pooled analysis of data from four studies. *Lancet.* (2016) 388:465–75. 10.1016/S0140-6736(16)30467-6 27216139

[27] Ma Y He FJ Sun Q Yuan C Kieneker LM Curhan GC 24-hour urinary sodium and potassium excretion and cardiovascular risk. *N Engl J Med.* (2022) 386:252–63. 10.1056/NEJMoa2109794 34767706 PMC9153854

[28] Zhuang P Zhang Y He W Chen X Chen J He L Dietary fats in relation to total and cause-specific mortality in a prospective cohort of 521 120 individuals with 16 years of follow-up. *Circ Res.* (2019) 124:757–68. 10.1161/CIRCRESAHA.118.314038 30636521

[29] Mazidi M Mikhailidis DP Sattar N Toth PP Judd S Blaha MJ Association of types of dietary fats and all-cause and cause-specific mortality: a prospective cohort study and meta-analysis of prospective studies with 1,164,029 participants. *Clin Nutr.* (2020) 39:3677–86. 10.1016/j.clnu.2020.03.028 32307197

[30] Zong G Li Y Wanders AJ Alssema M Zock PL Willett WC Intake of individual saturated fatty acids and risk of coronary heart disease in US men and women: two prospective longitudinal cohort studies. *BMJ.* (2016) 355:i5796. 10.1136/bmj.i5796 27881409 PMC5121105

[31] Lee JH Duster M Roberts T Devinsky O. United States dietary trends since 1800: lack of association between saturated fatty acid consumption and non-communicable diseases. *Front Nutr.* (2021) 8:748847. 10.3389/fnut.2021.748847 35118102 PMC8805510

[32] Salas-Salvado J Becerra-Tomas N Garcia-Gavilan JF Bullo M Barrubes L. Mediterranean diet and cardiovascular disease prevention: what do we know? *Prog Cardiovasc Dis.* (2018) 61:62–7. 10.1016/j.pcad.2018.04.006 29678447

[33] Mattioli AV Palmiero P Manfrini O Puddu PE Nodari S Dei Cas A Mediterranean diet impact on cardiovascular diseases: a narrative review. *J Cardiovasc Med (Hagerstown).* (2017) 18:925–35. 10.2459/JCM.0000000000000573 28914660

[34] Rosato V Temple NJ La Vecchia C Castellan G Tavani A Guercio V. Mediterranean diet and cardiovascular disease: a systematic review and meta-analysis of observational studies. *Eur J Nutr.* (2019) 58:173–91. 10.1007/s00394-017-1582-0 29177567

[35] Filippou CD Tsioufis CP Thomopoulos CG Mihas CC Dimitriadis KS Sotiropoulou LI Dietary approaches to stop hypertension (DASH) diet and blood pressure reduction in adults with and without hypertension: a systematic review and meta-analysis of randomized controlled trials. *Adv Nutr.* (2020) 11:1150–60. 10.1093/advances/nmaa041 32330233 PMC7490167

[36] Chiu S Bergeron N Williams PT Bray GA Sutherland B Krauss RM. Comparison of the DASH (Dietary approaches to stop hypertension) diet and a higher-fat dash diet on blood pressure and lipids and lipoproteins: a randomized controlled trial. *Am J Clin Nutr.* (2016) 103:341–7. 10.3945/ajcn.115.123281 26718414 PMC4733264

[37] Salehi-Abargouei A Maghsoudi Z Shirani F Azadbakht L. Effects of dietary approaches to stop hypertension (DASH)-style diet on fatal or nonfatal cardiovascular diseases–incidence: a systematic review and meta-analysis on observational prospective studies. *Nutrition.* (2013) 29:611–8. 10.1016/j.nut.2012.12.018 23466047

[38] Siervo M Lara J Chowdhury S Ashor A Oggioni C Mathers JC. Effects of the dietary approach to stop hypertension (DASH) diet on cardiovascular risk factors: a systematic review and meta-analysis. *Br J Nutr.* (2015) 113:1–15. 10.1017/S0007114514003341 25430608

[39] Panizza CE Shvetsov YB Harmon BE Wilkens LR Le Marchand L Haiman C Testing the predictive validity of the healthy eating index-2015 in the multiethnic cohort: is the score associated with a reduced risk of all-cause and cause-specific mortality? *Nutrients.* (2018) 10:452. 10.3390/nu10040452 29621192 PMC5946237

[40] Reedy J Lerman JL Krebs-Smith SM Kirkpatrick SI Pannucci TE Wilson MM Evaluation of the healthy eating index-2015. *J Acad Nutr Diet.* (2018) 118:1622–33. 10.1016/j.jand.2018.05.019 30146073 PMC6718954

[41] Parastouei K Sepandi M Eskandari E. Predicting the 10-year risk of cardiovascular diseases and its relation to healthy diet indicator in Iranian military personnel. *BMC Cardiovasc Disord.* (2021) 21:419. 10.1186/s12872-021-02231-y 34482840 PMC8419937

[42] Herforth AW Wiesmann D Martinez-Steele E Andrade G Monteiro CA. Introducing a suite of low-burden diet quality indicators that reflect healthy diet patterns at population level. *Curr Dev Nutr.* (2020) 4:nzaa168. 10.1093/cdn/nzaa168 33344879 PMC7723758

[43] Hariri N Darafshi Ghahroudi S Nasseri E Bondarianzadeh D Houshyar-Rad A Zayeri F. Evaluation of the alternative healthy eating index as a predictor of 10-year cardiovascular disease risk in a group of Iranian employees. *J Hum Nutr Diet.* (2017) 30:499–505. 10.1111/jhn.12416 27726209

[44] McCullough ML Feskanich D Stampfer MJ Giovannucci EL Rimm EB Hu FB Diet quality and major chronic disease risk in men and women: moving toward improved dietary guidance. *Am J Clin Nutr.* (2002) 76:1261–71. 10.1093/ajcn/76.6.1261 12450892

